# Evaluating UAV and Handheld LiDAR Point Clouds for Radiative Transfer Modeling Using a Voxel-Based Point Density Proxy

**DOI:** 10.3390/s26020590

**Published:** 2026-01-15

**Authors:** Takumi Fujiwara, Naoko Miura, Hiroki Naito, Fumiki Hosoi

**Affiliations:** 1 Department of Computer Science, National Defense Academy of Japan, Yokosuka 239-8686, Japan; ftakumi@nda.ac.jp; 2 Graduate School of Agricultural and Life Sciences, The University of Tokyo, Tokyo 113-8657, Japan; miura@uf.a.u-tokyo.ac.jp (N.M.); naito@g.ecc.u-tokyo.ac.jp (H.N.)

**Keywords:** canopy structure, point cloud, occlusion, near-infrared, correlation analysis, SLAM

## Abstract

The potential of UAV-based LiDAR (UAV-LiDAR) and handheld LiDAR scanners (HLSs) for forest radiative transfer models (RTMs) was evaluated using a Voxel-Based Point Density Proxy (VPDP) as a diagnostic tool in a *Larix kaempferi* forest. Structural analysis-computed coverage gap ratio (CGR) revealed distinct behaviors. UAV-LiDARs effectively captured canopy structures (10–45% CGR), whereas HLS provided superior understory coverage, but exhibited a high upper-canopy CGR (>40%). Integrating datasets reduced the CGR to below 10%, demonstrating strong complementarity. Radiative transfer simulations correlated well with Sentinel-2 NIR reflectance, with the UAV-LiDAR (r = 0.73–0.75) outperforming the HLS (r = 0.64–0.69). These results highlight the critical importance of upper-canopy modeling for nadir-viewing sensors. Although integrating HLS data did not improve correlation due to the dominance of upper-canopy signals, structural analysis confirmed that fusion is essential for achieving volumetric completeness. A voxel size range of 50–100 cm was identified as effective for balancing structural detail and radiative stability. These findings provide practical guidelines for selecting and integrating LiDAR platforms in forest monitoring, emphasizing that while aerial sensors suffice for top-of-canopy reflectance, multi-platform fusion is requisite for full 3D structural characterization.

## 1. Introduction

Understanding the structure and functioning of forest ecosystems relies heavily on remote sensing technologies. Among these, Light Detection and Ranging (LiDAR) has become an indispensable tool for characterizing three-dimensional (3D) forest structures and estimating above-ground biomass [1]. Recent advances in compact LiDAR systems—particularly unmanned aerial vehicle (UAV)-based LiDAR (UAV-LiDAR) and handheld LiDAR scanners (HLSs)—have dramatically improved the efficiency and spatial resolution of forest measurements. Unlike conventional terrestrial laser scanning (TLS), which requires static and time-consuming ground-based measurements, UAV-LiDAR and HLS systems enable dynamic scanning while in motion, allowing for rapid acquisition of dense point clouds over large areas. These platforms have been successfully used to estimate forest attributes such as tree height, canopy structure, diameter at breast height (DBH), and tree-level growth metrics [2,3,4,5,6,7]. HLS excels at capturing detailed understory and stem structures from ground level [8,9], whereas UAV-LiDAR provides efficient coverage of upper-canopy layers across extensive areas [10]. Furthermore, integrating these complementary platforms has been shown to enhance the accuracy of structural parameter estimation [11,12] and improve forest road mapping [13]. Consequently, UAV-LiDAR and HLSs have emerged as promising tools for operational forest inventory and timber resource monitoring [14].

High-resolution forest models derived from LiDAR data are not only valuable for inventory and structural assessments, but also serve as essential inputs for radiative transfer models (RTMs), which simulate the propagation of light within forest canopies [15]. The three-dimensional representation of canopy structure strongly influences RTM performance and is a critical determinant of model accuracy [16,17]. RTM-based simulations are widely used for the large-scale retrieval of forest biophysical parameters from satellite observations, establishing LiDAR as a potential bridge between ground-based measurements and spaceborne data [18]. Traditionally, high-precision forest structural models used in RTMs have been constructed from multiple scans of terrestrial laser scanning (TLS) data, as most conventional RTMs require accurate three-dimensional distributions of leaf area density (LAD) for simulating light attenuation [19,20]. However, in dense or structurally complex forests—such as those with a high leaf area index (LAI) and substantial understory occlusion—TLS data alone are often insufficient. In such cases, combining TLS with aerial LiDAR data has been shown to improve structural completeness and simulation accuracy [21]. Moreover, recent studies have demonstrated that the integration of UAV-LiDAR data with Sentinel-2 imagery enables the highly accurate estimation of above-ground biomass, further validating the potential of UAV-based LiDAR as an effective tool for large-scale forest biophysical assessments [22]. Compared to TLS, UAV-LiDAR and HLS platforms offer more flexible and cost-effective alternatives, enabling repeated and spatially extensive acquisitions across seasons and sites. Despite these advantages, the application of these mobile LiDAR systems in RTMs remains limited, primarily due to the challenges in accurately estimating LAD from point clouds affected by inertial measurement unit (IMU) errors and larger beam footprints. Consequently, the extent to which these datasets can serve as reliable inputs for RTMs has not been thoroughly investigated.

To address the challenges associated with LAD estimation and the associated error propagation, a simplified RT modeling framework based on a Voxel-based Point Density Proxy (VPDP) [23] was adopted. This approach uses the number of LiDAR points within each voxel as a proxy for light attenuation, thereby bypassing the need for explicit LAD calculation. The model assumes that point density is statistically proportional to the effective surface area interacting with incident radiation, allowing for canopy light attenuation to be represented in a data-driven manner rather than through explicit and often uncertain biophysical parameters. VPDP offers two major advantages: (1) it eliminates the dependency on labor-intensive LAD derivation, reducing the risk of parameter-induced errors masking structural differences and (2) it enables the use of diverse LiDAR datasets—including those from UAV and handheld platforms—for radiative modeling. By calibrating the model’s empirical parameters to maximize correlation with satellite observations, this framework isolates the structural fidelity of the point cloud as the primary variable. Consequently, the correlation coefficient becomes a direct metric for evaluating how well the LiDAR-derived geometry captures the actual light–canopy interactions, serving as a diagnostic tool for point cloud completeness. Initial studies have demonstrated strong agreement between simulated and satellite-observed canopy reflectance (correlation coefficient r = 0.78), indicating that this simplified model can reproduce realistic canopy radiative behavior even without explicit biophysical parameters. However, the accuracy of this framework is inherently sensitive to the structural fidelity of the input point clouds. When point density or spatial completeness is insufficient, the simulated radiative response can deviate substantially, highlighting the critical role of LiDAR data quality in model reliability. Furthermore, voxel size represents a critical parameter that must be carefully determined to enhance the characterization of forest structural properties [24]. The optimal voxel spatial resolution depends on multiple factors, including the plot size, the specifications of the employed LiDAR system, and the intrinsic structural complexity of the forest canopy [25].

Building on this concept, the primary objective of this study is to evaluate the suitability of UAV-LiDAR and HLS derived point clouds for radiative transfer simulations using VPDP. Rather than focusing solely on the physical accuracy of the model itself, we assess how differences in LiDAR sensor specifications, platform stability, and scanning geometry influence the model’s radiative estimates. Specifically, we investigate two key aspects: (1) how the LiDAR platform type (UAV vs. Handheld) and individual sensor models affect the density and vertical distribution of the generated 3D forest point clouds, and (2) how variations in these point cloud characteristics impact the accuracy and robustness of RT simulations.

By quantitatively linking LiDAR data quality with RTM performance, this study aims to establish a methodological foundation for applying emerging UAV-LiDAR and HLS in forest radiative transfer modeling. The findings are expected to provide practical guidelines for selecting and integrating LiDAR platforms in future large-scale forest monitoring and optical modeling applications.

## 2. Materials

### 2.1. Study Site

**Figure 1:**
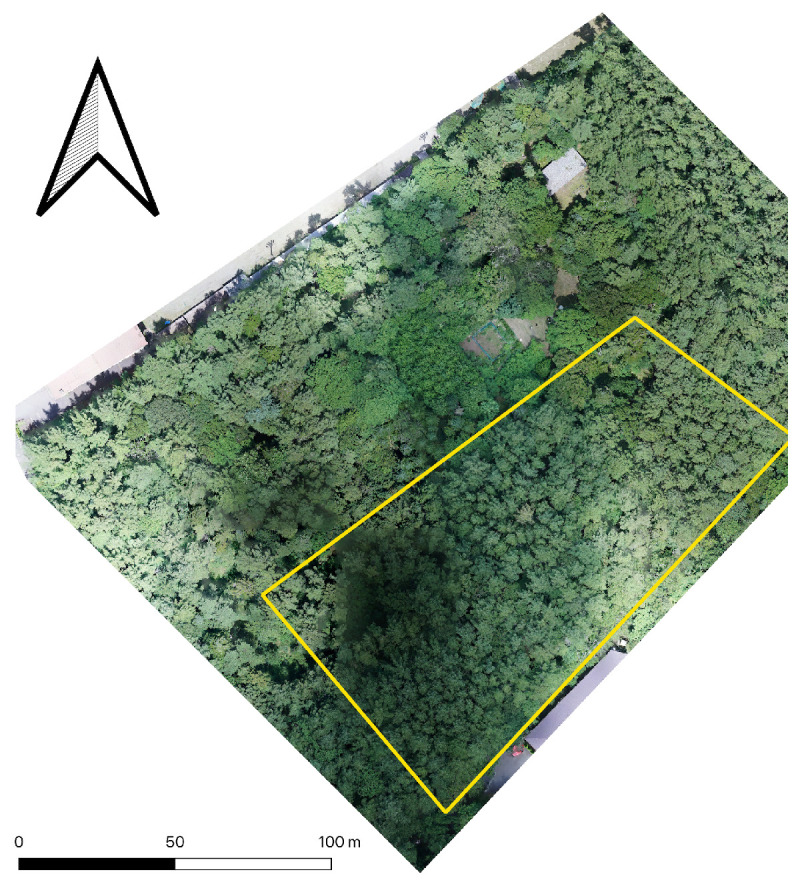
The study site location and radiative transfer simulation area (yellow).

This study was conducted within the Fuji Iyashinomori Woodland Study Center, the same research area utilized by Fujiwara et al. [23]. The site is situated at the foot of Mount Fuji in Yamanashi prefecture, Japan, at an approximate elevation of 1000 m (35°24′35″N,138°51′36″E). Ecologically, the area belongs to the upper cool-temperate zone. Although the broader region at the foot of Mount Fuji exhibits significant relief, the specific study plot is located on a gentle slope. Analysis using a 10 m Digital Elevation Model (Geospatial Information Authority of Japan, Tsukuba, Japan) indicated a mean slope of 3.0° and a mean aspect of 101°. Given this relatively flat topography, topographic correction for radiative transfer modeling was deemed unnecessary. The forest was originally established as a larch (*Larix kaempferi*) plantation in the 1920s. While larch remains the dominant species in the upper canopy, the forest now contains a mixed structure with various broadleaf species populating the sub-canopy and shrub layers. A 2019 survey reported a tree density of approximately 730 trees ha^−1^, with a mean DBH of 21.7 cm (range: 3.2–56.9 cm). For the present study, we established a 150 m × 70 m plot within a larch-dominant section of this site (Figure 1).

### 2.2. LiDAR Sensors and Data Acquisition

Four different LiDAR sensors were employed to capture forest structural information: two UAV-LiDAR (Explorer and Voyager, YellowScan SAS, Saint-Clément-de-Rivière, France) and two HLSs (Hovermap-ST, Emesent Pty Ltd., Milton, QLD, Australia; Trion S1, FJD Dynamics, Singapore). The specifications of each LiDAR sensor, including range accuracy, point density, and field of view, are summarized in Table 1 and Table 2. UAV-LiDAR data were acquired on 3 August 2023, at a flight altitude of 120 m above the same forest plot using a DJI Matrice 600 Pro platform. The Matrice 600 Pro (SZ DJI Technology Co., Ltd., Shenzhen, China) is a battery-powered multirotor UAV weighing 10 kg (including six batteries) and is capable of carrying up to a 6 kg payload. It is equipped with a D-RTK GNSS system, allowing for centimeter-level flight control accuracy. The Explorer and Voyager sensors were operated sequentially along the identical flight trajectory to ensure comparable observation geometry. Handheld LiDAR data were collected from the ground using the SLAM (Simultaneous Localization and Mapping) technique. The Hovermap-ST and TrionS1 measurements were performed along nearly identical walking paths to achieve the highest possible consistency in coverage. The SLAM algorithms are proprietary to each manufacturer and not publicly disclosed. All LiDAR datasets were georeferenced in a common coordinate system based on RTK-GNSS control points established within the study area. Only isolated outlier points were manually removed. No ground-surface extraction or automated filtering was applied in order to maintain the original point cloud characteristics and ensure comparability among sensors. Data acquisition was conducted under clear-sky and low-wind conditions to minimize motion-induced noise.

### 2.3. Sentinel-2 Image

The satellite imagery used in this study was obtained from the Sentinel-2 constellation. We employed the Top-of-Atmosphere (TOA) reflectance product and utilized the near-infrared (NIR) band (central wavelength: 842 nm; spatial resolution: 10 m). The imagery was retrieved from the Google Earth Engine platform using the harmonized Sentinel-2 dataset (Product ID: COPERNICUS/S2_HARMONIZED).

Although TOA reflectance contains atmospheric effects, it was selected for three methodological reasons: First, the analysis in this study is based on correlation rather than absolute reflectance values; therefore, systematic atmospheric biases do not affect the interpretation as long as they remain spatially consistent within the study area. Second, within a relatively small forested region, atmospheric conditions can be assumed to be uniform across the scene, minimizing within-scene variability introduced by using TOA instead of surface reflectance. Third, Bottom-of-Atmosphere (BOA) reflectance produced by atmospheric correction algorithms is known to introduce inconsistencies due to interactions among vegetation density, terrain slope, and spectral band characteristics. These inconsistencies are particularly pronounced in mountainous vegetated areas, where substantial discrepancies between TOA and BOA reflectance have been reported for the red-edge and near-infrared bands [26]. For these reasons, TOA reflectance was considered more appropriate for maintaining internal consistency and avoiding terrain- and vegetation-dependent correction artifacts.

## 3. Methods

**Figure 2:**
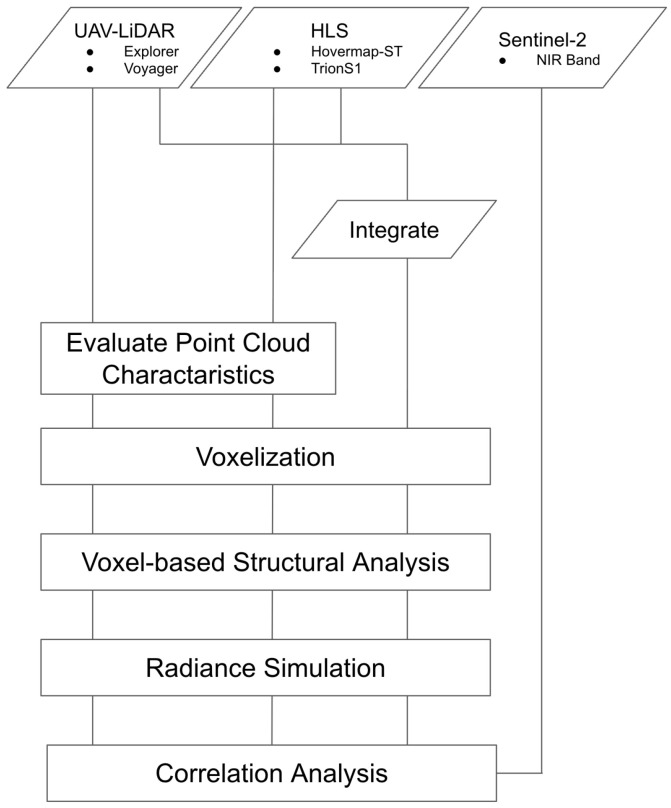
Overall research flowchart.

Figure 2 illustrates the methodological workflow of this study. Point clouds acquired from the UAV-LiDAR and HLSs were first visualized using cross-sectional and planar views to reveal their structural characteristics. Subsequently, these point clouds, along with their integrated datasets, were converted into voxel representations, which were then used for a simplified structural analysis. Next, relative radiance was simulated using the VPDP RT framework, and the resulting irradiance maps were resampled to a 10 m resolution to align with the Sentinel-2 grid. Finally, the simulated radiance was compared with the Sentinel-2 NIR reflectance to evaluate the performance of each point cloud dataset.

### 3.1. Voxel-Based Point Density Proxy Modeling Framework

**Figure 3:**
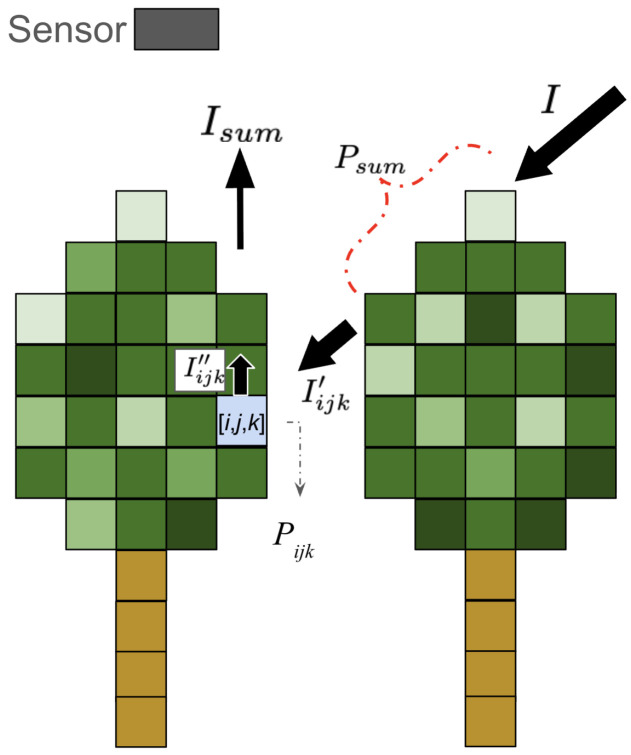
Incident irradiance *I* is attenuated by canopy voxels according to the Beer–Lambert law, where voxel-wise LiDAR point counts are used as a proxy for leaf area density. The transmitted irradiance $I_{ijk}^{\prime }$ and upward radiance $I_{ijk}^{\prime \prime }$ are computed using Equations (1)–(3), and total column radiance $I_{sum}$ is obtained by integrating radiance contributions along the sensor direction.

The essential steps of VPDP [23] are summarized here. The attenuation of direct irradiance within the canopy was modeled using the Beer–Lambert law, where the cumulative number of LiDAR points along the light path was used as a proxy for LAD. Figure 3 provides an overview of the simulation concept. The shading of each voxel represents the number of LiDAR points contained within it.

After the incident irradiance *I* is attenuated by the canopy, the transmitted irradiance reaching voxel [i,j,k], denoted as $I_{ijk}^{\prime }$, is computed using Equation (1). Here, $P_{sum}$ represents the cumulative number of LiDAR points stored in the voxels located between the light source and [i,j,k], and $\alpha (point^{-1})$ is an empirical coefficient converting point density into a light attenuation factor. The upward radiance $I_{ijk}^{\prime \prime }$ from voxel [i,j,k] toward the sensor is calculated based on a Lambertian reflection assumption using Equation (2), where $\beta (point^{-1})$ is an additional attenuation coefficient analogous to α. R is the leaf spectral reflectance. The total radiance for each column is then aggregated using Equation (3). Finally, the simulated radiance map is resampled to a 10 m spatial resolution using average aggregation so that it matches the pixel resolution of the Sentinel-2 imagery. The two empirical parameters, α and β, were optimized independently for each LiDAR dataset to maximize the Pearson correlation between the simulated and observed Sentinel-2 TOA reflectance in the NIR band. This optimization process functions as a scene-specific radiometric calibration, where α serves to normalize the differences in point density and return sensitivity among sensors. By statistically aligning the simulated radiometric magnitude with the satellite observation, this approach ensures that the spatial variance in the simulated irradiance is primarily driven by the voxelized canopy structure. Consequently, the resulting correlation coefficient reflects the structural fidelity of the input point cloud rather than the accuracy of absolute radiative transfer parameterization.

(1) $$I_{ijk}^{\prime }=Iexp\left(-\alpha P_{sum}\right)$$

(2) $$I_{ijk}^{\prime \prime }=R\frac{I_{ijk}^{\prime }\exp\;\left(-\beta \sum_{k=k+1}^{n}P_{ijk}\right)}{\pi }$$

(3) $$I_{sum}=\sum_{k=0}^{N}I_{ijk}^{\prime \prime }$$

### 3.2. Evaluation Metrics

The performance of each LiDAR sensor was evaluated through two complementary approaches: (1) geometric and structural assessment based on point cloud and voxel analysis, and (2) radiometric assessment using RT simulation.

#### 3.2.1. Point Cloud and Voxel-Based Evaluation

First, the geometric characteristics of the point clouds were evaluated using cross-sectional and planar density maps. These visualizations revealed the horizontal and vertical acquisition characteristics of each LiDAR system. Next, point clouds derived from each LiDAR sensor, as well as their combined datasets (Explorer–HovermapST, Explorer–TrionS1, Voyager–HovermapST, and Voyager–TrionS1), were converted into voxel models with four voxel sizes (20 cm, 50 cm, 100 cm, and 200 cm). Each voxel stored the number of points contained within it. The voxelized data were vertically stratified into four layers (0–10 m, 10–20 m, 20–30 m, and 30–40 m above the minimum Z-coordinate), and the median point count per voxel was computed for each layer to visualize the vertical distribution of point density.

To evaluate coverage gaps, each voxel model was compared against a reference dataset generated by merging all four LiDAR-derived point clouds (hereafter referred to as the all-merged voxel). Let $V_{All}$ denote the set of voxels in the all-merged dataset and $V_{c}$ denote the set of voxels derived from an individual or combined LiDAR dataset. The set of voxels that exist only in $V_{All}$ but not in $V_{c}$, denoted as $V_{All-only}$, was computed as shown in Equation (4). Subsequently, for each height layer *z*, the coverage gap ratio ($CGR_{z}$) was defined as the proportion of voxels present only in $V_{All}$ relative to the total number of voxels in $V_{All}$ at that height, as shown in Equation (5).

The voxel overlap ratio between each dataset and the all-merged voxel model was calculated for every height layer. Although complete occlusion-free modeling in dense forests is practically unattainable, the all-merged voxel dataset was assumed to represent an almost complete structural model of the study site. This comparison enabled a quantitative assessment of how effectively each LiDAR sensor, or a combination of sensors, captured the forest structure relative to the integrated reference dataset.

(4) $$V_{\mathrm{All}\text{-}\mathrm{only}}=\left\{\left(i,j,k\right)|\left(i,j,k\right)\in V_{All}\wedge \left(i,j,k\right)\notin V_{c}\right\}$$

(5) $$CGR_{z}=\frac{|V_{\mathrm{All}\text{-}\mathrm{only}\text{-}z}|}{|V_{All-z}|}$$

#### 3.2.2. RT Simulation-Based Evaluation

The second evaluation metric assessed the radiometric performance of each point cloud when used as input to the RT simulation. Specifically, the simulated radiance was compared with Sentinel-2 TOA NIR reflectance using the Pearson correlation coefficient. While RT simulations are often validated using absolute error metrics such as RMSE, this study focused on the reproducibility of spatial reflectance patterns as a more fundamental indicator. Although canopy-induced shading is not the sole determinant of satellite-observed reflectance, regions exhibiting stronger shading generally correspond to lower reflectance values, and this shading pattern is inherently linked to voxel-wise point density. Therefore, the correlation between simulated and observed reflectance was treated as a proxy for assessing the structural fidelity of the LiDAR-derived 3D models. Pixels used for comparison with Sentinel-2 reflectance were manually screened to exclude non-target species, as the study site is not composed exclusively of *Larix kaempferi* and the RT model was parameterized using its spectral properties. In Equation (1), the incident irradiance I was set to 1 because the analysis relied on relative correlation rather than absolute magnitude. The leaf reflectance parameter R in Equation (2) was set to 0.05, representing the mean NIR reflectance of four *Larix kaempferi* spectra from the library [27].

Considering the geometric accuracy of Sentinel-2 (2.5 m; ref. [28]), the simulated radiance maps were horizontally shifted by up to ±3 m in both X and Y directions. The shift that produced the maximum correlation coefficient was adopted as the final evaluation result.

To strictly assess the predictive power of the point clouds and rule out overfitting due to parameter optimization, we performed a random subsampling validation. The dataset was randomly split into a calibration set (70%) and a validation set (30%). Parameters (α and β) were optimized using the calibration set, and the Pearson correlation coefficient was calculated using the independent validation set. This process was repeated 20 times with different random seeds to ensure statistical robustness, and the mean and standard deviation of the correlation coefficients were reported.

## 4. Results

### 4.1. Cross-Sectional and Planar Visualization of Point Clouds

**Figure 4:**
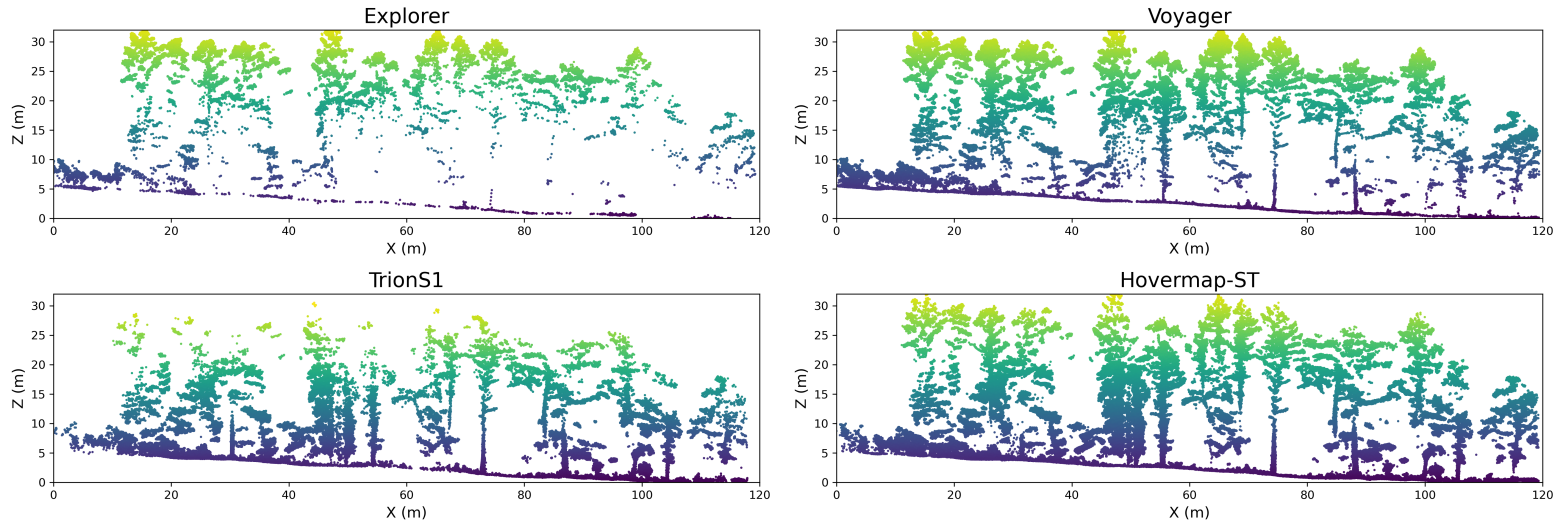
Cross-sectional comparison of point clouds obtained from the four LiDAR systems. Each transect represents a 100 m × 1 m section of the study plot, color-coded by elevation.

To complement the planar and vertical density analyses, a cross-sectional transect (approximately 100 m wide and 1 m deep) was extracted from each LiDAR dataset to visually assess the spatial distribution of canopy and ground returns (Figure 4). The color scale represents elevation. For the UAV-LiDARs, Voyager exhibited dense point returns across both the canopy and ground layers, clearly capturing tree stems and understory structures. In contrast, Explorer primarily recorded upper-canopy points with limited penetration into the lower layers. Among the HLSs, Hovermap-ST successfully not only captured the understory, but also a portion of the upper canopy, indicating a broader vertical scanning capability. TrionS1 produced lower point density in the upper canopy, but adequately covered the ground and lower canopy layers. These results highlight the influence of sensor type, scanning geometry, and SLAM-based trajectory accuracy on the completeness of point cloud acquisition within forest environments.

**Figure 5:**
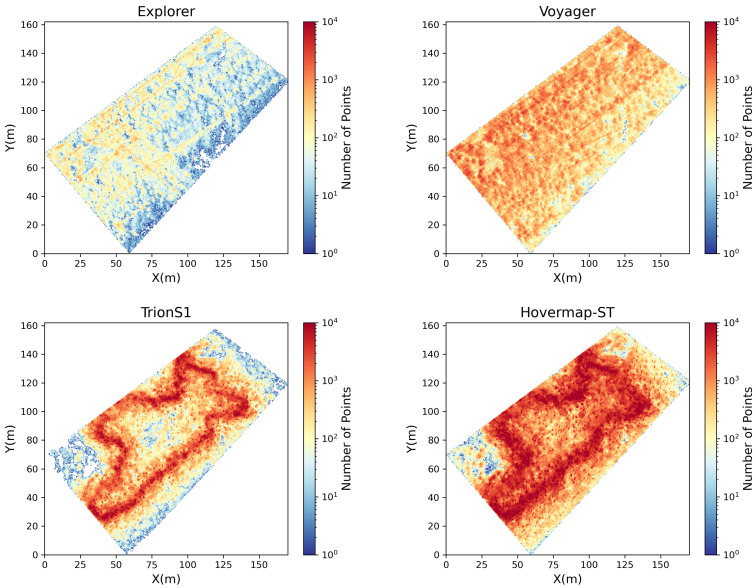
Spatial distribution of point cloud density (number of points per 0.5 m grid) for four LiDAR sensors: UAV-LiDARs (Explorer and Voyager) and HLSs (TrionS1 and Hovermap-ST). Warmer colors indicate higher point densities on a logarithmic scale. Each sensor exhibits distinct spatial coverage and density gradients reflecting differences in scanning geometry and platform motion.

Figure 5 shows planar maps of point cloud density generated using a 0.5 m grid for each LiDAR sensor. For the UAV-LiDAR systems, Voyager provided a nearly uniform point distribution across the entire forest area, indicating consistent flight performance and scanning overlap. In contrast, Explorer exhibited strong directional bias: high-density regions were observed along the primary flight paths, whereas areas between scan lines showed more than an order of magnitude lower density. Notably, around X = 100 m and Y = 70 m, Voyager successfully captured canopy structures, while Explorer exhibited gaps in data coverage. For the HLSs, both Hovermap-ST and TrionS1 showed higher point densities along operator walking trajectories. TrionS1 exhibited a dense coverage zone within approximately 10 m from the trajectory (up to 8000 points per grid), followed by a rapid decrease in density with distance. Hovermap-ST, on the other hand, maintained a relatively high density up to 15–20 m from the trajectory, suggesting wider effective scanning coverage. Around X = 25 m and Y = 70 m, Hovermap-ST detected high-density points even away from the main trajectory, indicating its ability to capture canopy surfaces beyond the immediate path, likely due to a wider scanning field or stronger return sensitivity. Overall, these results highlight the influence of platform stability, scanning geometry, and sensor configuration on point density distribution—factors crucial for subsequent 3D forest structure modeling.

### 4.2. Voxel-Based Structural Analysis

**Figure 6:**
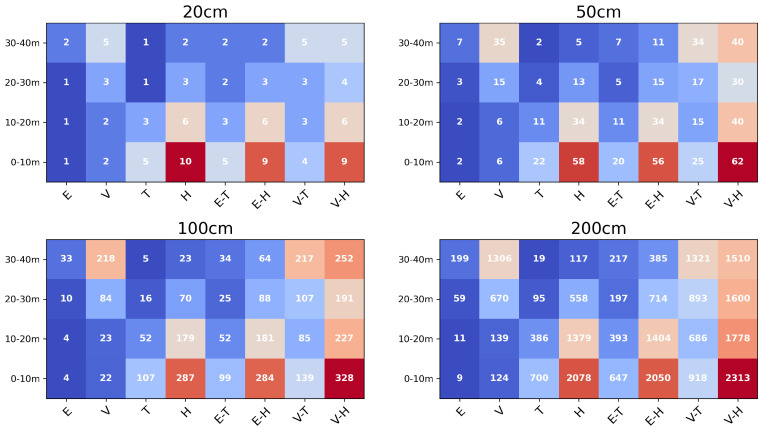
Vertical distribution of the median number of points for different voxel sizes (20 cm, 50 cm, 100 cm, and 200 cm). The point cloud was divided into 10 m height intervals (0–10 m, 10–20 m, 20–30 m, and 30–40 m) from the lowest Z-coordinate, and the median point density was computed for each layer. Each column represents the results of individual sensors (E: Explorer, V: Voyager, T: TrionS1, H: Hovermap-ST) and their integrated datasets.

Figure 6 illustrates the vertical distribution of point cloud density across four voxel sizes (20 cm, 50 cm, 100 cm, and 200 cm). For the UAV-LiDAR sensors, Voyager and Explorer, the difference in density became more pronounced as voxel size increased. At the 20 cm voxel size, Voyager exhibited approximately twice the density of Explorer, while at 50 cm, the difference widened to about fivefold. In the lowest layer (0–10 m), Voyager averaged around two points per voxel at 20 cm resolution, increasing to about six points at 50 cm, whereas Explorer remained around two points. For the HLSs, Hovermap-ST achieved a higher density than TrionS1 across all height layers. At 50 cm voxel size, Hovermap-ST recorded an average of 13 points in the 20–30 m layer and 5 points in the 30–40 m layer. TrionS1, in comparison, showed approximately one-third of those densities, reflecting its narrower scanning field and lower effective range. When the UAV-LiDAR and handheld LiDAR datasets were combined, the Voyager–HovermapST (VH) combination consistently produced the highest density across all height layers and voxel sizes. The Explorer–TrionS1 (ET) combination, however, showed limited improvement: at voxel sizes of 20 cm and 50 cm, the density in the 30–40 m layer remained identical to Explorer alone (2 and 7 points, respectively), suggesting minimal synergy in canopy capture. Across all configurations, the 20–30 m height layer showed consistently lower density compared to other layers, indicating a structurally sparse region likely corresponding to the mid-canopy zone. These findings demonstrate how sensor type, voxel resolution, and data fusion strategy collectively influence the vertical completeness of 3D LiDAR representations, which is essential for accurate forest radiative transfer modeling.

**Figure 7:**
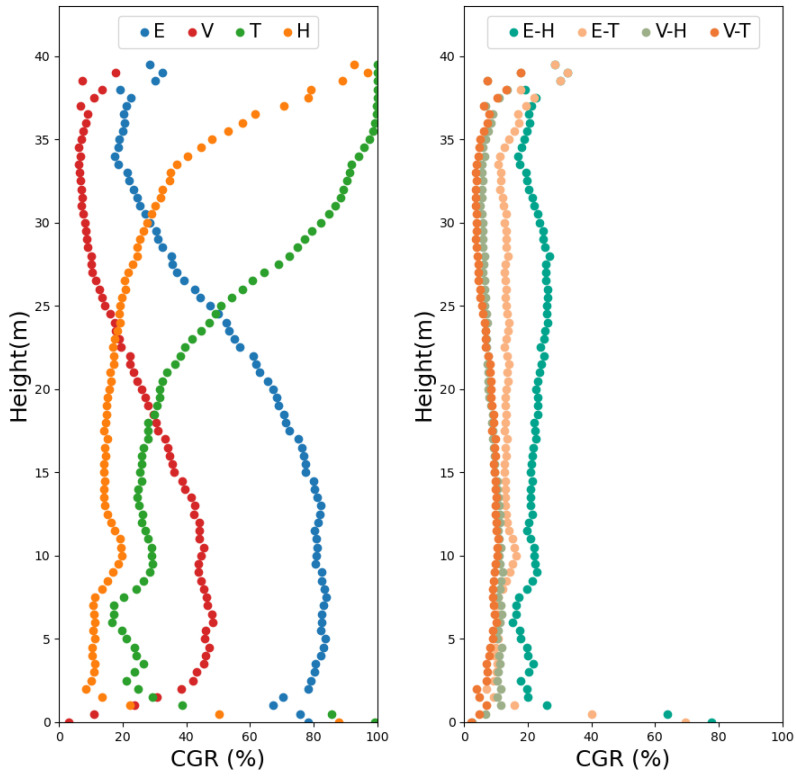
Vertical profiles of the coverage gap ratios (CGRs) for each LiDAR dataset compared with the all-merged voxel reference model. (**Left**) Single-sensor point clouds (E: Explorer; V: Voyager; T: TrionS1; H: Hovermap-ST) and (**Right**) Combined datasets (E-H: Explorer–HovermapST; E-T: Explorer–TrionS1; V-H: Voyager–HovermapST; V-T: Voyager–TrionS1). The X-axis represents the mean CGR within each 50 cm vertical layer and the Y-axis denotes height above ground level.

Figure 7 illustrates the vertical distribution of CGR, defined as the proportion of voxels in the all-merged model that were not represented in each individual or combined point cloud. A higher CGR indicates a greater degree of occlusion. The left panel shows the results for single-sensor datasets, while the right panel presents those for combined UAV–handheld LiDAR datasets. Because the 20 cm voxel size occasionally contained voxels with only a single point (particularly in sparse canopy layers), a 50 cm voxel size was adopted for the gap analysis to ensure statistical stability. The Y-axis represents the height above the lowest voxel level, and the X-axis denotes the mean CGR for each 50 cm vertical bin. For the UAV-LiDAR sensors, the CGR of Voyager ranged from a minimum of 10% (30–35 m) to a maximum of 45% (5–13 m). Explorer exhibited a broader range, with a minimum of 20% (34–36 m) and a maximum of 80% (5–13 m). In both cases, CGR increased sharply below 25 m, and the difference between Voyager and Explorer widened with decreasing height. At canopy heights (30–40 m), their CGR difference was approximately 10–20%, whereas below 25 m, it reached 20–40, reflecting the greater susceptibility of Explorer to low-canopy shadowing. For the HLSs, Hovermap-ST maintained a relatively low CGR (20) up to 25 m, but showed a sharp increase to 45 between 25 and 33 m, followed by a further rise near the upper canopy. TrionS1, on the other hand, exhibited 20 CGR below 7 m, which increased to 40 between 7 and 20 m and rose steeply above that height.

When the UAV-LiDAR and handheld LiDAR datasets were integrated, CGR decreased substantially across all height layers. The Voyager–HovermapST and Voyager–TrionS1 combinations produced nearly identical results, maintaining CGRs below 10 above 3 m. Only the Explorer–TrionS1 combination exhibited local peaks exceeding 20 below 3 m height. These results demonstrate that the fusion of UAV- and HLS point clouds effectively compensates for the CGR effects inherent to individual sensor configurations, particularly within the lower- and mid-canopy layers.

### 4.3. Correlation Between Simulated Irradiance and Sentinel-2 NIR Reflectance

**Figure 8:**
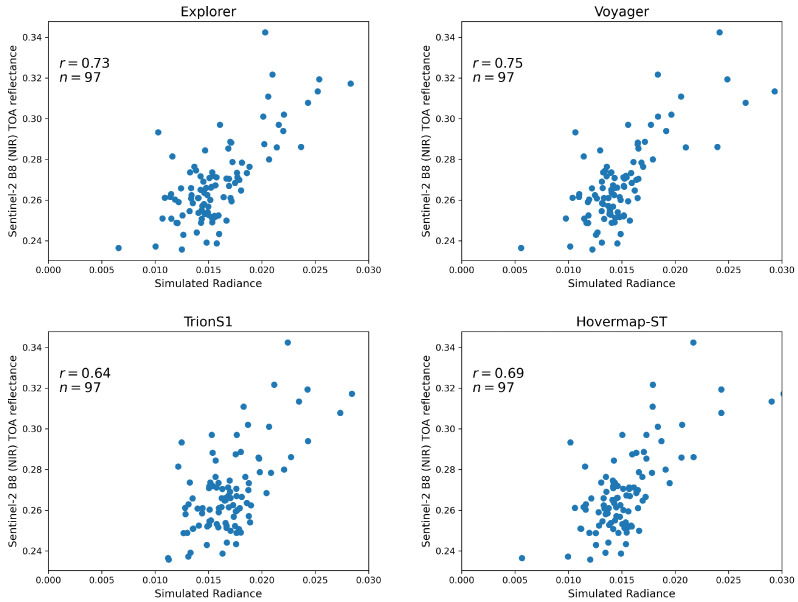
Correlation between simulated radiance derived from individual LiDAR point clouds and Sentinel-2 NIR (Band 8) TOA reflectance.

Figure 8 summarizes the correlation analysis between the simulated radiance derived from each individual sensor’s point cloud and the Sentinel-2 NIR (Band 8) TOA reflectance. A voxel size of 50 cm was adopted for this comparative analysis. Based on the vertical point density distributions shown in Figure 6, this resolution was identified as the essential common baseline to ensure a fair inter-sensor comparison. Specifically, 50 cm represents the minimum resolution required for the lowest-density sensor (Explorer) to achieve sufficient voxel-wise point counts, thereby ensuring statistical stability while preserving high structural fidelity for the higher-density sensors (Voyager and HLS). Out of the initial 100 Sentinel-2 pixels covering the study area, 3 were excluded based on the screening criteria, resulting in a final sample size of 97 (n = 97) available for comparison. Among the four sensors, Voyager exhibited the highest correlation (r = 0.75), followed by Explorer, Hovermap-ST, and TrionS1. This ranking corresponds to the order of point-cloud abundance in the 30–40 m canopy layer at a voxel size of 50 cm (see Figure 4). The difference in r between the two UAV-LiDAR systems, Voyager and Explorer, was minimal (0.02). In contrast, Hovermap-ST alone achieved a relatively high correlation (r = 0.69), whereas TrionS1 showed the lowest value (r = 0.64). The robustness of these results was confirmed through repeated validation analysis. Table 3 summarizes the mean and standard deviation of the correlation coefficients obtained from the 20 validation iterations (30% hold-out data). The mean correlation values (e.g., r = 0.73 for Explorer and r = 0.74 for Voyager) closely match the correlations derived from the full dataset shown in Figure 8. This consistency demonstrates that the VPDP model retains high predictive capability for independent data and that the optimization process did not result in overfitting.

The dynamic range of the simulated irradiance also differed across sensors. Voyager, Explorer, and Hovermap-ST produced a similar range of approximately 0.025, while TrionS1 yielded a narrower range of about 0.017. Notably, the minimum simulated value for TrionS1 was 0.012, whereas the other cases reached approximately 0.005, indicating that TrionS1 failed to capture the low-radiance points observed by the other sensors.

**Figure 9:**
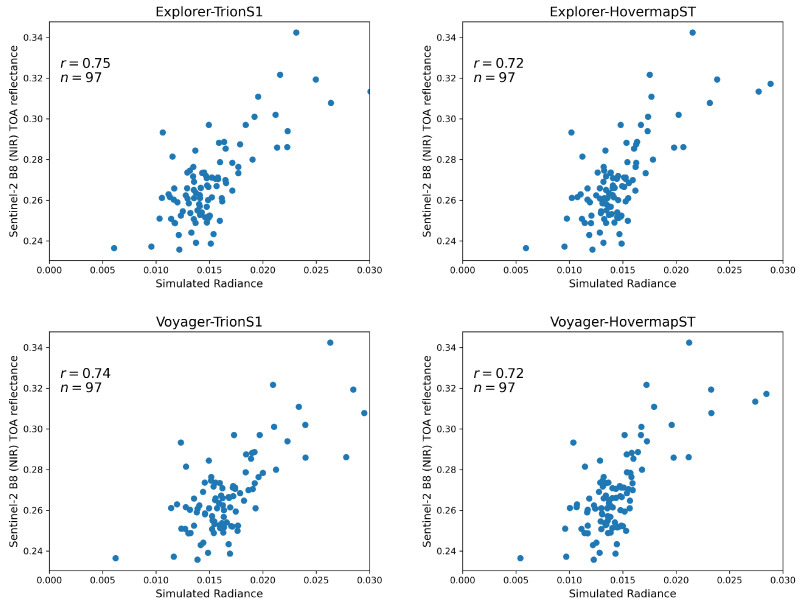
Correlation between simulated radiance derived from integrated LiDAR point clouds (UAV-LiDAR + HLS combinations) and Sentinel-2 NIR (Band 8) TOA reflectance.

Figure 9 shows the results obtained when point clouds from the UAV-LiDAR and HLS were combined prior to the radiative transfer simulation. The combination of sensors produced little change in the correlation coefficients, which remained within a narrow range (0.72–0.75). The difference in the dynamic range of simulated radiance was small across all combinations (minimum: 0.05; maximum: 0.03). The predictive robustness of these integrated datasets was further verified through the repeated validation analysis summarized in Table 4. The mean correlation coefficients ranged from 0.69 to 0.75, closely matching the results derived from the full dataset.

**Figure 10:**
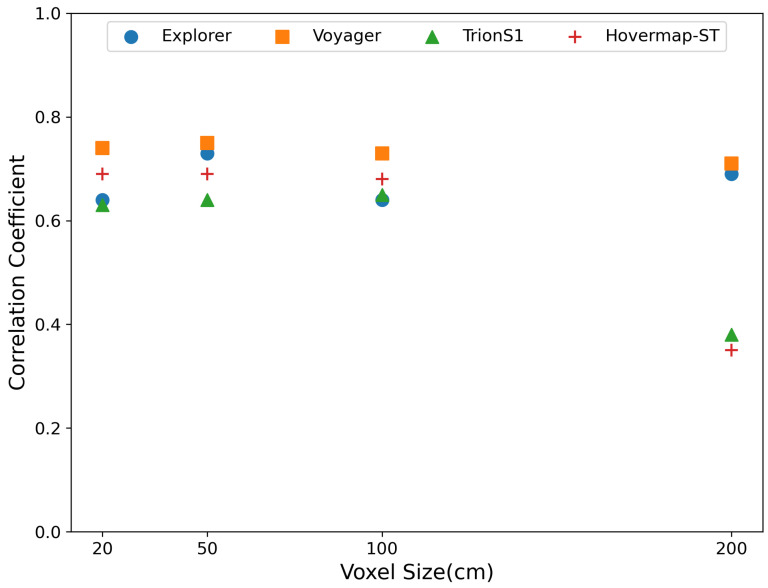
Relationship between voxel size (20, 50, 100, and 200 cm) and correlation coefficient (r) between simulated radiance and Sentinel-2 NIR reflectance for each LiDAR sensor. UAV-LiDAR (Explorer and Voyager) maintained relatively high and stable correlations across voxel sizes, whereas HLSs (TrionS1 and Hovermap-ST) showed a marked decline in correlation at coarsest voxel resolution (200 cm).

Figure 10 shows the relationship between voxel size (20, 50, 100, and 200 cm) and the correlation coefficient between simulated irradiance and Sentinel-2 NIR reflectance for each individual LiDAR sensor. Among the UAV-LiDAR systems, Voyager maintained the highest and most stable correlation values across all voxel sizes, ranging from 0.71 to 0.75. Explorer exhibited its lowest correlation at 20 cm (r = 0.64), followed by an increase at 50 cm (r = 0.73) and 100 cm (r = 0.74) and a slight decrease at 200 cm (r = 0.69). For the handheld LiDAR sensors, both TrionS1 and Hovermap-ST showed nearly constant correlation values from 20 to 100 cm (approximately 0.64 and 0.69, respectively). However, their performance dropped markedly at 200 cm, with correlation coefficients decreasing by approximately 0.3 compared with finer voxel resolutions. Overall, the UAV-LiDAR systems achieved higher and more consistent correlations than the handheld LiDAR sensors across all voxel sizes.

## 5. Discussion

### 5.1. Sensor-Specific Structural Representation and Complementarity

The comparative analysis of UAV- and handheld-based LiDAR systems revealed distinct sensor-specific characteristics in forest structure representation, consistent with previous multi-platform studies [25,29]. UAV-LiDARs provided superior canopy coverage, but exhibited substantial occlusion in the understory, while HLS captured detailed ground and understory features but lacked sufficient canopy penetration. These complementary acquisition behaviors are well aligned with earlier findings [21,30]. However, the present study extends these insights by quantitatively examining CGR profiles using voxel-based metrics (Figure 7). Despite both being UAV-LiDAR, Voyager and Explorer demonstrated markedly different vertical CGR patterns. Voyager achieved the lowest overall CGR (10–45%) across the canopy, while Explorer exhibited up to 80% CGR near the lower canopy (5–13 m). This difference can be attributed to Voyager’s higher return multiplicity (32 returns versus five in Explorer) and greater point generation rate (525 vs. 50 pts/m^2^), as summarized in Table 1. Among the HLSs, Hovermap-ST demonstrated greater vertical reach and a broader scanning envelope than TrionS1, maintaining a low CGR (20%) up to 25 m. In contrast, TrionS1 exhibited a rapid CGR increases above 7 m, reaching 40% by 20 m. These discrepancies reflect the influence of hardware configurations such as pulse rate, SLAM accuracy, and field of view (360° × 290° for Hovermap-ST vs. 360° × 270° for TrionS1).

Importantly, the integration of the UAV-LiDAR and HLS datasets substantially reduced the CGR across all height layers, indicating strong complementarity between aerial and terrestrial viewpoints. Both Voyager–HovermapST and Voyager–TrionS1 combinations achieved nearly identical occlusion profiles, maintaining <10% CGR above 3 m, suggesting that high-performance handheld LiDARs can effectively compensate for UAV-LiDAR blind zones regardless of aerial sensor type. In contrast, the Explorer–TrionS1 combination, representing lower-specification sensors on both platforms, still exhibited >20% CGR in some layers, emphasizing the critical role of sensor performance in achieving structural completeness.

It is important to acknowledge that the all-merged voxel model used as a reference for the CGR calculation is derived from the same set of UAV and handheld sensors and does not represent an absolute ground truth verified by TLS or destructive sampling. Consequently, the calculated CGR represents the relative completeness of each sensor compared to the integrated dataset rather than absolute field occlusion. While the fusion of aerial and terrestrial viewpoints effectively minimizes blind spots—potentially exceeding TLS coverage in the upper canopy—future research should incorporate independent high-precision validation data (e.g., multi-scan TLS) to rigorous quantify the absolute physical consistency of the voxelized structure.

Recent advances in low-cost, consumer-grade LiDARs have expanded opportunities for 3D vegetation modeling, including orchard and plantation studies [31]. While such systems offer economic advantages, their integration into forest-scale analysis remains challenging due to limited range, reduced accuracy, and restricted return multiplicity. The present findings suggest that, even for high-performance systems, vertical completeness is sensitive to sensor capability. Consequently, the integration of heterogeneous LiDAR data—ranging from TLS, UAV-LiDAR, and handheld SLAM-based systems to photogrammetric point clouds (Structure-from-Motion, SfM)—requires rigorous voxel-based harmonization to ensure structural fidelity [19].

### 5.2. Influence of Sensor Characteristics on Radiative Transfer Simulation Accuracy

The correlation analysis between simulated irradiance and Sentinel-2 NIR reflectance (Figure 8) revealed that the UAV-LiDAR systems achieved higher accuracies (r = 0.73–0.75) than the HLSs (r = 0.64–0.69). These values indicate a generally strong agreement, comparable to or exceeding previous studies. For instance, Cao et al. [32] simulated hyperspectral canopy reflectance using the LESS model [33] and reported correlation coefficients of r = 0.56–0.59 in the NIR region. Although the simulation targets differ, the present LAD-free framework demonstrated comparable or superior radiometric consistency, underscoring its potential for representing canopy light interactions. It should be noted that the high correlations observed in this study are partly attributable to the optimization of the weighting parameters (α and β) against the reference satellite data. Ideally, such optimization on a limited dataset raises concerns about overfitting. However, our repeated validation analysis (20 iterations of 70% calibration/30% validation split) yielded a high mean correlation (r = 0.72) for independent test data, comparable to the full-dataset results. This empirical stability confirms that the optimization did not merely fit the noise, but successfully calibrated the radiometric magnitude. Thus, the remaining strong spatial correlation confirms that the VPDP model successfully translates the voxel-based structural variations into realistic radiometric patterns. This validates the premise that, when radiometric responses are calibrated, the remaining variance in simulation accuracy serves as a robust proxy for assessing the structural fidelity and completeness of the LiDAR point clouds without the need for complex LAD inversion.

Despite Voyager having over six times more return channels than Explorer (32 vs. 5 returns), the difference in correlation was relatively small (▵r = 0.02). This result suggests that, beyond a certain threshold, increasing the number of returns does not substantially enhance the reconstruction of canopy reflectance at satellite observation scales. A return capacity of approximately five may already suffice for capturing upper-canopy geometry critical for radiative transfer simulation. Regarding the HLSs, Hovermap-ST exhibited slightly lower but comparable correlations (r = 0.69) to UAV-LiDAR, whereas TrionS1 performed marginally lower (r = 0.64). Achieving UAV-equivalent simulation accuracy using only HLS data would require improved modeling of upper-canopy structures. In this study, the HLS data were collected along a single trajectory; multiple passes or multi-angle scanning could mitigate canopy occlusion effects, especially at heights above 25 m. Given the operational flexibility and ease of deployment of handheld systems, developing optimized acquisition strategies for RT modeling—particularly in broadleaved or mixed forests—represents an important avenue for future research.

Despite the structural complementarity achieved by integrating the UAV-LiDAR and HLS datasets, the correlation with Sentinel-2 NIR reflectance did not significantly improve compared to the UAV-only results. This is likely because Sentinel-2 is a nadir-viewing satellite sensor, meaning that its reflectance signals are predominantly influenced by the upper canopy layers. Consequently, the additional information captured by the HLS regarding the lower and understory structure contributed little to the nadir-based reflectance simulation. However, this does not diminish the value of data integration. The detailed understory geometry provided by the HLSs is essential for physically realistic RTM simulations, particularly for modeling light transmittance, gap fraction, and energy absorption within the canopy—factors that nadir reflectance alone cannot fully capture. For applications involving off-nadir observations, biomass estimation, or carbon stock assessment—contexts in which volumetric canopy characteristics play a larger role—the inclusion of HLS-derived lower-canopy information is expected to be critically important. Future work should therefore investigate how multi-angular satellite data or LiDAR–RT model integration can more effectively leverage the complementary structural information across canopy layers.

While this study prioritized the NIR band to evaluate the structural fidelity of point clouds, establishing a comprehensive RTM requires validation across broader spectral and temporal scales. Future work should therefore extend this framework to include multi-spectral (e.g., Red-Edge, SWIR) and multi-temporal analyses to capture phenological changes. Additionally, comparing the simulated results with atmospherically corrected surface reflectance and ground-based radiometric measurements will be essential for validating the absolute radiometric accuracy of the model.

### 5.3. Influence of Voxel Size on Radiative Transfer Simulation Accuracy

The effect of voxel size on the correlation coefficient was further examined using four voxel resolutions (20, 50, 100, and 200 cm). For Explorer, the correlation was lowest at the smallest voxel size (20 cm, r = 0.64), increased substantially at 50 cm (r = 0.73) and 100 cm (r = 0.74), and slightly decreased again at 200 cm (r = 0.69). This pattern suggests that excessively fine voxelization can amplify local irregularities in point distribution, while overly coarse voxelization may oversimplify important canopy structural features.

It is important to note that the 50 cm voxel size was selected as a common resolution for inter-sensor comparison. While optimizing voxel size individually for each sensor (e.g., using finer voxels for HLS) might theoretically maximize each sensor’s potential, varying the voxel size would introduce inconsistencies in the radiative transfer physics, hindering direct comparison. The 50 cm selection was driven by the lowest-density sensor (Explorer), which exhibited instability at finer resolutions (20 cm), but stabilized at 50 cm. Since higher-density sensors (Voyager and HLS) also maintained high performance at this resolution (Figure 10), 50 cm represents the effective “minimum common resolution” that allows for a fair benchmarking of all platforms under consistent geometric conditions.

Overall, these results indicate that voxel sizes of approximately 50–100 cm provide an optimal balance between preserving fine-scale canopy geometry and maintaining radiative stability. The absence of improvement at 20 cm can likely be attributed to increased point-cloud noise and spatial heterogeneity at very fine resolutions. For the HLSs, correlations dropped by roughly 0.3 at the coarsest 200 cm voxel size, consistent with the lower canopy point density and higher occlusion observed in Figure 6 and Figure 7.

Similar findings were reported by Liu et al. [34], who suggested that a voxel resolution of 1–2 m is optimal for calibrating and validating remote sensing products in temperate deciduous forests. Although forest type and canopy structure differ from the present study site, the computational trade-offs and stability patterns observed here support a comparable conclusion. Nevertheless, the optimal voxel resolution is also expected to depend on the spatial resolution of the target satellite sensor and the characteristic scale of the canopy elements (e.g., crown size, gap distribution). Therefore, the 50–100 cm range identified here should be interpreted as specific to the *Larix kaempferi* forest and Sentinel-2 configuration. Therefore, future research should systematically evaluate how voxel resolution influences RT simulation accuracy across different forest types and satellite observation scales.

## 6. Conclusions

This study evaluates the influence of LiDAR sensor performance on point-cloud characteristics and RT simulation accuracy in a *Larix kaempferi* forest using multiple UAV- and handheld-based LiDAR systems. Structural assessment based on planar density, vertical voxel distribution, and CGR analysis revealed clear sensor-dependent differences: UAV-LiDARs captured canopy structure more effectively, while handheld systems provided superior coverage of the understory. Integrating both platforms substantially reduced the CGR across height layers, confirming their complementary capabilities in reconstructing a complete 3D forest model.

By employing an empirically calibrated voxel-based RT framework (VPDP), this study demonstrates that simulation accuracy can serve as a robust proxy for evaluating the structural fidelity of LiDAR point clouds. The simulations showed strong correlations with Sentinel-2 NIR reflectance (r = 0.73–0.75 for UAV-LiDARs; r = 0.64–0.69 for HLS). Critically, the optimization of model parameters normalized the radiometric differences among sensors, confirming that the observed variations in correlation were primarily driven by the spatial quality of the input point clouds.

The analysis identified 50–100 cm as the optimal voxel size range for this specific forest environment, balancing canopy structural detail and radiative stability. Although the inclusion of handheld data did not significantly improve the correlation with nadir-viewing satellite imagery due to upper-canopy masking, the CGR analysis highlights that fusion is essential for applications requiring volumetric completeness, such as light transmittance modeling and biomass estimation.

In conclusion, the VPDP framework provides a practical, diagnostic approach for assessing LiDAR data quality. It offers a scalable method for bridging the gap between ground/aerial point clouds and satellite observations, providing valuable guidelines for future multi-sensor forest modeling and large-scale ecosystem monitoring.

## Figures and Tables

**Table 1:** Technical specifications of UAV-LiDAR (Explorer and Voyager).

Technical Specification	Explorer	Voyager
Accuracy (1σ@50 m, nadir)	2 cm	1 cm
Laser wavelength	1556 nm	1550 nm
Point density	50 pts/m^2^@100 m	525 pts/m^2^@120 m
	AGL 10 m/s	AGL 10 m/s
Number of Return	5	32
Laser range	Up to 500 m	Up to 1250 m

**Table 2:** Technical specifications of HLSs (TrionS1 and Hovermap-ST).

Technical Specification	Trion S1	Hovermap-ST
Sensing Range	120 m @ 90% reflectivity	0.40 to 100 m
	80 m @ 10% reflectivity	
Accuracy	2 cm	2 cm
Laser wavelength	905 nm	905 nm
FOV	360° × 270°	360° × 290°
Number of Return	1	3
Points Per Second	320,000	600,000 (Dual return)

**Table 3:** Validation results for individual LiDAR platforms. Mean Pearson correlation coefficients (r) and standard deviations were calculated from 20 iterations of stratified random subsampling (70% calibration/30% validation).

Sensor	Mean r	Standard Deviation
Explorer	0.73	0.090
Voyager	0.74	0.083
TrionS1	0.63	0.091
Hovermap-ST	0.68	0.098

**Table 4:** Validation results for combined LiDAR platforms (UAV + Handheld). Mean Pearson correlation coefficients (r) and standard deviations were calculated from 20 iterations of stratified random subsampling (70% calibration/30% validation).

Sensor Combination	Mean r	Standard Deviation
Explorer–TrionS1	0.74	0.078
Explorer–HovermapST	0.69	0.111
Voyager–TrionS1	0.75	0.058
Voyager–HovermapST	0.74	0.057

## Data Availability

The data are not publicly available.
